# Intermittent energy restriction for weight loss: Spontaneous reduction of energy intake on unrestricted days

**DOI:** 10.1002/fsn3.586

**Published:** 2018-02-21

**Authors:** Jennifer Harvey, Anthony Howell, Julie Morris, Michelle Harvie

**Affiliations:** ^1^ Knowsley Community Diabetes Service Aintree University Hospitals NHS Foundation Trust Liverpool UK; ^2^ Prevent Breast Cancer Research Unit Manchester University Hospital NHS Foundation Trust Manchester UK; ^3^ Department of Statistics Manchester University Hospital NHS Foundation Trust Manchester UK

**Keywords:** energy intake, energy restriction, food records, intermittent, weight loss

## Abstract

There is increasing interest for the use of intermittent energy restriction (IER) in weight management. However, there are concerns that IER could result in ‘rebound’ overconsumption of energy on unrestricted days. We studied self‐reported food records from participants in two trials of IER versus continuous energy restriction (Study 1; 44 women on IER for 6 months and Study 2; 72 women on two types of IER for 4 months). Energy intake was assessed on restricted and unrestricted days immediately before and after restricted days and on other unrestricted days. We assessed consistency of days of the week chosen as restricted days, and whether this was associated with greater weight loss. Reported energy intake was reduced on unrestricted days in Study 1 and 2 and was 19% lower compared with the allocated isoenergetic diet, and respectively 21% and 29% lower than their baseline reported daily intakes. Energy intake appeared to be similarly reduced the day immediately before and after restricted days and on other unrestricted days. Seventy percent of women in Study 1 and 79% in Study 2 undertook consistent days of restriction each week (>50% of restricted days on the same 2 days each week). When studies were combined percentage weight loss at 3 months was −5.8 (−6.7 to −4.7) % in the consistent group and −7.4 (−8.7 to −6.2) % in the non‐consistent group (*p* = .09). Food records from patients undertaking IER suggest a spontaneous reduction in energy intake below their baseline reported intakes and the prescribed isoenergetic diet during all unrestricted days including the days immediately before and after restricted days which contributes to the weight loss success with these diets. Consistency of restricted days was not associated with weight loss success. These findings need to be confirmed in larger groups of patients ideally using objective measures of energy balance.

## 1 INTRODUCTION

Obesity is a well‐established cause of premature mortality and ill‐health. In the United Kingdom (UK) alone the cost attributable to overweight and obesity to the National Health Service (NHS) was estimated to be £15.8 billion per year in 2007 (Public Health England, 2016). Consequently obesity management and the prevention of type 2 diabetes are major priorities for the NHS (NHS England, 2016, 2014). Limited success and problems of adherence to standard daily diets are well known (Anastasiou, Karfopoulou, & Yannakoulia, 2015) and thus, there is a need for novel, evidence based and cost‐effective strategies to support weight management.

There is increasing interest in the use of intermittent energy restriction (IER) for weight management and thus disease risk (Johnstone, 2014; Seimon et al., 2015). IER consists of periods of a marked energy restriction, typically either 60%–75% reduction below predicted energy requirements for 2 days each week, with 5 days of baselineintake (Harvie et al., 2010, 2013) or alternating days of 75% energy restriction below predicted energy requirements and normal eating (Varady et al., 2013; Bhutani et al., 2013; Klempel, Bhutani, Fitzgibbon, Freels, & Varady, 2010). Clinical trials show IER can lead to significant reductions in body weight and adiposity either comparable to or greater than continuous energy restriction (CER; Harvie et al., 2010, 2013; Varady et al., 2013; Bhutani et al., 2013; Klempel et al., 2010; Davis et al., 2016).

Despite evidence suggesting IER might be beneficial for weight loss in some individuals with overweight or obesity (Harvie et al., 2010, 2013; Varady et al., 2013; Bhutani et al., 2013; Klempel et al., 2010; Davis et al., 2016), there are concerns about the safety of IER and the potential for compensatory overeating on unrestricted days that is seen in animal models (Harvie & Howell, 2016) and some types of IER in humans (Laessle, Platte, Schweiger, & Pirke, 1996). In order to assess the extent of compensatory overeating on unrestricted days we re‐analysed energy intake in the IER arms of two randomised trials of comparisons of IER and CER (Harvie et al., 2010, 2013). Since overeating may potentially occur most on the days before and after the two restricted days we specifically assessed energy intake on these days in comparison with the other unrestricted days. Here we show that contrary to predictions of overeating there was a 19% spontaneous reduction in energy intake below their prescribed diet on all five unrestricted days in both studies.

## 2 METHOD

Dietary data were analysed from two previously published randomised trials of IER versus CER in overweight women previously reported by Harvie et al. (2010, 2013). Both of these studies were ethically approved; Study 1 (Harvie et al., 2010) by the South Manchester Ethics Committee (reference 05/Q1403/243) and Study 2 (Harvie et al., 2013) by the North Manchester Research Ethics Committee (reference 09/H1006/34).

### 2.1 Dietary interventions

Study 1 tested a 2 day IER in 53 premenopausal women with overweight or obesity (BMI 24–45 kg/m^2^) over six months. The IER was designed to provide an overall 25% energy restriction below estimated energy requirements and involved two consecutive days each week of 70% energy restriction below estimated energy requirements, and five relatively unrestricted days /week designed to meet 93% of their estimated daily energy requirements calculated using the Schofield Equation (Schofield, 1985). Restricted days in Study 1 provided 2,700 kJ and 50 g protein and comprised of 1.136 L (2 pints) of semi skimmed milk, four 80 g portions of vegetables, one portion of fruit, a salty low energy drink, and an over the counter multivitamin and mineral supplement. Normal eating days advised a Mediterranean‐type diet (30% of energy from fat; [7% saturated, 15% monounsaturated and 7% polyunsaturated fatty acids], 45% low glycaemic load carbohydrate and 25% protein). Participants were advised on maximal quantities of protein, carbohydrate and fat on these days and weekly guidance for treat foods (3 per week) and alcohol (14 units/week) to prevent over consumption. Seven day food diaries were completed after 1 and 6 months on the diets and were checked for completeness with participants and analysed using Compeat Nutritional Analysis System (Carlson Bengston Consultants, London, UK).

Study 2 tested two different versions of a 2 day IER for 3 months of weight loss and 1 month of weight maintenance (with one restricted day per week) amongst 75 pre and post menopausal women with overweight or obesity (BMI 24–45 kg/m^2^). One IER was designed to provide an overall 25% energy restriction below estimated energy requirements and involved two consecutive days of energy and carbohydrate restriction each week (energy restricted low carbohydrate IER, 2,500–2,717 kJ/day, <50 g carbohydrate/day). Each restricted day allowed approximately 250 g of protein foods, three servings of low‐fat dairy foods, four 80 g portions of low‐carbohydrate vegetables and one portion of low‐carbohydrate fruit, at least 1,170 ml of other low‐energy fluids, and an over the counter multivitamin and mineral supplement. The five unrestricted days were as above with advice on maximal portions of foods and treats to ensure participants did not overconsume. The other two day IER regimen tested was similar, but allowed unlimited lean meat, fish, eggs, tofu and unsaturated fats on restricted days (ad libitum low carbohydrate IER). Seven day food diaries were collected and checked for completeness with each participant at 1, 3 and 4 months and were analysed via Wisp (Tinuviel Software, Anglesey, Wales).

We determined energy intake on restricted and unrestricted days from available 7 day food diaries from the two studies. We report energy intake on the two restricted and the five unrestricted days and compare energy intake on unrestricted days immediately before and after restricted days compared with other unrestricted days to explore the presence of any energy compensation surrounding restricted days. Data were analysed using the Generalised Estimating Equation (GEE) regression model (with exchangeable correlation) using Wald chi‐square tests, with type of day (i.e. unrestricted day before or after restricted day or other unrestricted day), day of the week and month of food diary completed (i.e. month 1 vs. month 3) as main effects. Analyses were adjusted for the day of the week in view of the observed increase in energy intake previously reported over weekends (An, 2016).

In addition to 7 day food diaries participants in both trials were also asked to complete simple weekly records throughout the whole study period to indicate if they had undertaken their restricted days that week and the precise timing of restricted days during the week. When 50% or more of restricted days were undertaken on the same days each week subjects were classed as having consistent restricted days whilst participants with less than 50% of restricted days undertaken on the same day were classed as having inconsistent restricted days. We compared percentage weight loss between women with consistent versus inconsistent restricted days each week across both studies using an independent *T* test. Data were analysed using SPSS (Version 22, SPSS Limited).

## 3 RESULTS

We included subjects allocated to IER in the two trials who had completed a baseline at least one 7 day food diary during the IER period. This included both successful (≥5% weight loss) and unsuccessful (<5% weight loss) dieters from both trials. Study 1 included 44 subjects (83% of the original IER cohort). Thirty‐nine subjects (89%) had 7 day food diaries available for analysis at 1 month and 20 (45%) had dietary records at 6 months. Consistency of restricted days chosen during the 6 month weight loss period could be measured in 38 subjects, 72% of the original IER cohort. Study 2 included 72 subjects, 36 following the energy restricted low carbohydrate IER and 36 the ad libitum low carb IER (96% of the IER cohort). Sixty‐five subjects (90%) had 7 day food diaries available for analysis at 1 month, 55 subjects (76%) at 3 months and 49 subjects (68%) at 4 months. Consistency of restricted days chosen during the 3 month weight loss period could be measured in 69 subjects, 92% of the original IER cohort.

Characteristics of the populations included in this analysis are shown in Table 1. Both studies included women, many of whom had a family history of breast cancer, were mainly white British with median (interquartile range) 2 (1–5) previous attempts to lose weight. Sixty‐three percent of participants from Study 1 and from Study 2 within both IER groups successfully lost weight (≥5% weight loss), whilst 37% from both studies were not successful using this criterion.

**Table 1:** Baseline characteristics and weight change of the subjects in Study 1 and Study 2 included in this analysis

	Study 1 (*n* 44)	Study 2 (*n* 72)
Age (years)^a^	39.7 (4.3)	47.3 (8.0)
BMI (kg/m^2^)^a^	31.1 (5.3)	30.1 (5.0)
Body fat (%)^a^	40.7 (5.5)	38.7 (5.2)
LOCF % weight loss at 3 months^a^	7.0 (4.5)	6.0 (4.2)
LOCF % weight loss at 4 months^a^	Not measured	6.7 (4.9)
LOCF % weight loss at 6 months^a^	8.8 (6.1)	Not measured
Family history of breast cancer (%)		
Yes	50	100
No	50	0
Employment (%)		
Full time	86.4	68.1
Part time	9.1	19.4
Retired or unemployed	4.5	12.5
Menopausal status (%)		
Pre/perimenopausal	100	55.2
Post	0	44.8
Children living at home (%)		
Yes	77	65
No	23	35
Ethnicity (%)		
White British	93.2	98.6
Other	6.8	1.4
Smoker, *n* (%)	0	6 (8)
Previous attempts at dieting^b^	2 (1–5)	2 (1–5)

BMI, body mass index; LOCF, last observation carried forward.

a Mean (SD).

b Median (interquartile range).

### 3.1 Self reported adherence to the restricted days within IER

The cohorts included in this analysis reported good adherence to IER. In Study 1 the mean (95% CI) percentage self reported adherence to the potential two restricted days per week during the six month trial period was 85 (79–81) %. In Study 2 adherence to the potential restricted days (i.e. two per week for 3 months and one day per week in the fourth month) was 75 (67–85) % for the energy restricted low carbohydrate IER and 77 (69–87) % for the ad lib low carbohydrate IER. All participants included from Study 1 completed their two restricted days on consecutive days as shown by food records. In Study 2 90% of participants routinely did their two restricted days consecutively, with the remainder as separate days within the week. In Study 1 mean (95% CI) energy intake on restricted days was 2,966 (2,761–3,176) kJ/day; in Study 2 this was 2,895 (2,769–3,016) kJ/day for the energy restricted low carbohydrate IER group and 4,318 (4,062–4,577) kJ/day for the ad lib low carbohydrate IER group.

### 3.2 Energy intake on unrestricted days of IER

Subjects in both studies were advised to consume a Mediterranean‐type diet that met 93% of their estimated energy requirements on the five unrestricted days of the week. In Study 1 and Study 2 the recommended intakes were respectively 4% and 6% less than their reported baseline energy intake (Table 2). In Study 1 the mean (95% CI) recommended intake during unrestricted days was 7,728 (7,536–7,921) kJ, whereas actual mean (95% CI) intake was 6,250 (5,937–6,564) kJ/day, indicating 19% restriction on these days. In Study 2 the mean (95% CI) recommended intake during unrestricted days for both groups was 7,546 (7,399–7,691) kJ, whereas the actual mean intakes were comparable between the energy restricted low carbohydrate IER 5,775 (5,243–6,243) kJ/day and the energy restricted ad lib low carbohydrate IER 6,427 (5,907–6,945) kJ/day (*p* > .05). The combined groups in Study 2 had a 19% energy restriction below the recommended intake on these days. Thus the overall energy restriction across the whole week including restricted and unrestricted days in Study 1 and 2 was 39% below estimated energy requirements, rather than the planned 25% energy prescription.

**Table 2:** Energy intake at baseline and on the different unrestricted days of intermittent energy restriction in Study 1 (*n* = 44) and Study 2 (*n* = 67) in (kJ/day)

	Baseline reported intake	Recommended intake on unrestricted day, 93% of estimated energy requirements	Day immediately before restricted day	Other un‐restricted day	Mean (95% CI) difference between days immediately before and other unrestricted day	*p* value Day immediately before and other unrestricted day	Day immediately after restricted day	Other un‐restricted day	Mean (95% CI) difference between days immediately after and other unrestricted day	*p* value Day immediately after and other unrestricted day
Study 1 energy intake (kJ/day)( )*n *=* *44	7,928 (7,397, 8,458)	7,728 (7,536–7,921)	6,226 (5,799, 6,648) (59 days)^a^	6,230 (5,866, 6,594) (177 days)^a^	−4 (−381, 368)	.98	6,427 (5,966, 6,883) (59 days)^a^	6,226 (5,841, 6,607) (177 days)^a^	201 (−335, 736)	.46
Study 2 energy intake (kJ/day) *n *=* *67	8,484 (8,049, 8,923)	7,546 (7,399–7,691)	5,925 (5,535, 6,318) (73 days)^a^	6,117 (5,807, 6,427) (632 days)^a^	−192 (−506, 121)	.23	6,042 (5,665, 6,418) (166 days)^a^	6,134 (5,820, 6,443) (632 days)^a^	−92 (−393, 209)	.55

Mean (95% CI).

Mean values for energy intake on the day “immediately before” and the day “immediately after” restricted days and “other unrestricted” day are adjusted means values derived from the separate regression models which adjusted for day of the week.

a Total number of days assessable.

### 3.3 Energy intake on days immediately before and after restricted days compared to other unrestricted days

In Study 1, data were available on 44 subjects for 59 days immediately following (after) and 59 days immediately before a restricted day (before) and for 177 other unrestricted days (other) not including days adjacent to a restricted day. Using GEE regression analysis, no statistical significant differences were detected in energy intake on the day immediately ‘after’ the two restricted days compared with the ‘other’ unrestricted days (*p* = .46). After adjusting for the day of the week there was no significant difference across the months that food diaries were completed (*p* = .38), and no significant difference between days of the week (*p* = .09). There were no statistical significant differences in energy intake between “before” and “other” unrestricted days (*p* = .98), after adjusting for the day of the week and no significant difference between days of the week (*p* = .08) nor between months (*p* = .78).

In Study 2, data were available on 67 subjects for 166 days immediately after and 73 immediately before a restricted day and for 632 other unrestricted days not including days adjacent to a restricted day. Again by GEE regression there were no statistically significant differences in energy intake between ‘after’ restricted days and ‘other’ days (*p* = .55), after adjusting for the day of the week and no significant difference between months (*p* = .70). Likewise there was no statistical significant differences in energy intake between ‘before’ and ‘other’ days (*p* = .23), after adjusting for the day of the week and no significant difference between months (*p* = .53). These findings are summarised in Table 2.

### 3.4 Days of the week chosen for restricted days

Weekdays were the most commonly chosen as days for restriction, although a minority of subjects in Study 2 chose to start their weekly two restricted days at the weekend (5% on a Saturday and 2% on a Sunday; Table 3).

**Table 3:** Days of the week chosen by subjects as the first weekly restricted day during Study 1 and Study 2

First restricted day	Study 1 (59 weeks) *n* (%)	Study 2 (168 weeks) *n* (%)
Monday	26 (44)	37 (22)
Tuesday	7 (12)	29 (17)
Wednesday	15 (25)	36 (21)
Thursday	6 (10)	47 (28)
Friday	5 (8)	6 (4)
Saturday	0 (0)	9 (5)
Sunday	0 (0)	4 (2)

In Study 1 the percentage of subjects classed as choosing consistent restricted days (i.e. chose the same restricted days ≥50% of the weeks) was 74%. The remaining 26% had more variable patterns. In Study 2 79% were classed as consistent, and the remaining 21% had more variable patterns.

Weight loss was comparable between women with consistent versus non‐consistent restricted days. When studies were combined mean (95% CI) percentage weight loss at 3 months was −5.8 (−6.7 to −4.7) % in the consistent group (*n* = 84) and −7.4 (−8.7 to −6.2%) (*n* = 23) in the non‐consistent group (*p* = .09).

## 4 DISCUSSION

Contrary to concerns of compensatory energy increase on unrestricted days this self‐ report data finds an apparent relatively large spontaneous 19% reduction in energy intake below the allocated diet on unrestricted days, and below their baseline reported energy intake before commencing the diet. This apparent ‘carry over effect’ was seen across all unrestricted days in the food records including the days immediately before and after restricted days and is likely to be an important component contributing to the overall energy deficit and efficacy of IER for weight loss.

There are few reports of adherence to IER and energy compensation on unrestricted days of IER in the literature. Klempel et al. (2010) reported a modest (5%) reduction in energy intake on unrestricted days of IER amongst subjects with obesity undertaking 8 weeks of alternate days of a 75% energy restriction below estimated energy requirements and ad libitum intake following the National Cholesterol Education Program dietary guidelines. The lack of compensation of energy intake in our trial of subjects following an IER for weight loss is interesting and contrasts to previous short term fasting studies conducted in laboratory settings amongst subjects not attempting to lose weight. Studies amongst subjects with overweight or obesity have reported a compensatory 10%–23% increase in energy intake from their baseline intake on the day following a single day of either a 25% energy restriction or a total fast (Antoni, Johnston, Collins, & Robertson, 2016). Whilst Johnstone et al. reported a 20% increased energy intake on the day after a 36 hr total fast amongst 12 lean men and 12 lean women (Johnstone et al., 2002). Laessle et al. (1996) reported a large energy compensation and hyperphagia (40% increased intake) on unrestricted days when nine healthy weight women (mean BMI of 21.1 ± 1.5 kg/m^2^) undertook 4 weeks of IER with four consecutive days of 2,510 kJ and three unrestricted days of ad libitum feeding each week. The women in this study were classified as unrestrained eaters, and they were not attempting to lose weight, thus their drive to eat and behaviours are likely to be different to that in overweight and obese subjects following IER with the aim of weight loss.

The reason for the spontaneous reduction in energy intake on unrestricted days of IER in our studies deserves further investigation. Reduced energy intake could be due to behavioural aspects of following an IER as part of a weight loss programme. Anecdotal reports from subjects in the two reported trials suggest IER can make individuals more aware of their habitual food intakes and habits, increase awareness of appetite and hunger, and provide assurance that they can function adequately during restricted days without the need for extra energy on surrounding days. Bhutani et al. (2013) reported increased restrained eating and decreases in uncontrolled eating amongst overweight subjects undertaking alternate day energy restriction (75%).

The effects of IER on hunger and appetite are not well defined. We and others have reported that hunger scores have been found to be elevated during restricted days in the initial weeks of IER given for weight loss, and to either reduce over time, suggesting habituation to the regime (Harvie et al., 2013; Bhutani et al., 2013; Klempel et al., 2010), or remain increased (Heilbronn, Smith, Martin, Anton, & Ravussin, 2005). Some studies have reported decreased feelings of fullness on restricted days of IER which have been reported either to normalise over time (Varady et al., 2013; Bhutani et al., 2013; klempel et al., 2010; heilbronn et al., 2005) or to remain low throughout the 8 week IER intervention. To our knowledge there are no published data of hunger and fullness on unrestricted days of IER regimens.

The hormones leptin and ghrelin influence energy intake, decreased leptin and increased ghrelin both increase appetite (Klok, Jakobsdottir, & Drent, 2007). Serum leptin has been reported to decrease on both restricted and unrestricted days when following an IER diet (Harvie et al., 2010, 2013; Varady et al., 2013). We have reported increased ghrelin on restricted and unrestricted days of IER (Harvie et al., 2010), however, studies of alternate day fasting amongst patients who are non‐obese (Heilbronn et al., 2005) or obese (Johnson et al., 2007) have not shown changes in ghrelin on either restricted or unrestricted days of the IER regimen. Recent studies have reported energy restriction to evoke increased appetite despite reduced ghrelin levels but increased hormone sensitivity (O'Connor et al., 2016). Thus more detailed studies of changes in appetite‐mediating hormones levels and sensitivity with IER are required.

The lack of energy compensation in our study is perhaps to be expected as we had counselled participants not to overeat by advising healthy meals and portion guidance on unrestricted days. However, reported energy intake is below their allocated diet on these unrestricted days which was not advised. The lack of energy compensation on unrestricted days in our studies may not occur if IER diets are presented as spells of severe restriction and spells of feasting as is the case with many commercial IER diets.

Many subjects chose consistent restricted days each week. Following a routine may help to form habits and ultimately contribute to dietary adherence (Gardner, Sheals, Wardle, & McGowan, 2014). However, consistent days did not associate with better weight loss, and may reflect the fact that subjects varied their restricted days to aid dietary compliance and to accommodate their lifestyle. Most restricted days were undertaken on weekdays, however, a minority chose weekend days, perhaps because this would allow more time to plan and prepare for the diet.

Strengths of the current study include that the participants were following the IER diets for weight loss in a ‘real world’ situation. These data provides a better reflection of eating behaviours when people are following IER compared to short term fasting experiments conducted in laboratory settings. The participants in both diet studies were self‐selecting meals so these findings are likely to be replicable amongst people following IER for weight loss in other clinical and non‐clinical settings.

The analysis included a significant proportion of subjects from the two IER trials (83% of the IER group from Study 1 and 86% of the IER groups from Study 2), and included women who were ultimately successful and less successful with weight loss (≥ and <5% weight loss). However average intakes reported herein are derived from all available records in these cohorts during their period of IER. Some women contributed 2 or 3 diaries whilst others contributed one only, in some cases as they subsequently withdrew from the trial. We only used available data and did not make assumption about dietary intake where food records were not availaible. We acknowledge that the food records reported herein are likely to reflect a best case scenario and weeks when participants were most compliant with IER. Research dietitians ensured food diaries were checked for completeness with subjects during the trial to maximise their accuracy. Food diaries were carefully coded with the days of the week. This enabled us to adjust the analysis for day of the week, thus removing any confounding effects of variability in energy intake over the week and higher intakes at weekends which have been previously reported amongst the general population (An, 2016).

We have found that reduced intake on unrestricted days is replicated with three different IER regimes which were tested in two different populations which varied in terms of age and menopausal status, suggesting that this may be a general response to IER with two consecutive days of restriction.

A major limitation of the analysis is that the data were derived from self‐reported 7 day food diaries which are well known to under report dietary intake (Subar et al., 2015). However, it is assumed that if underreporting did take place it would have been across all food diaries, across time points, including the baseline diary. Importantly intake on non‐restricted days was reported to be 21%–29% lower than their baseline intake.

The observed weight loss seen in these Study 1 and 2 cohorts were respectively 5.7 and 5.9 kg at 3 months. Modelling the reported energy deficit using the national Institute of Health Weight Loss modelling programme (National Institutes of Health) would predict losses of around 7 kg in both cohorts suggesting a degree of underreporting. However re‐running the models with the assumption that subjects were only undertaking their reported restricted days each week and did not reduce intake on the other days would only predict 50% of the observed weight loss, indicating that a significant proportion of the weight loss achieved with IER is achieved through spontaneous reduction of energy intake on the non‐restricted days.

We have reported energy intake and not commented on the quality of the diet throughout unrestricted days. Participants were encouraged to follow a Mediterranean diet on unrestricted days, in line with healthy eating practices. They achieved a healthy diet on unrestricted days in terms of macronutrient composition i.e. monounsaturated fat, and fibre which has previously been reported within these published studies (Harvie et al., 2010, 2013).

This report suggests intermittent diets may be a useful strategy to promote weight loss, without the concern for caloric compensation. However, further research is needed in longer term intermittent dieting regimes, and in diverse populations including men, and amongst normal weight subjects following IER with an aim of improving their health rather than attempting to lose weight. The observation of a spontaneous reduction in energy intake below their prescribed diet on unrestricted days is of interest. These findings from self reported food records need validation with more objective measures of energy balance. Assuming the spontaneous reduction is a real phenomenon of IER, the mechanism warrants further behavioural and physiological research to better understand how these diets work.

## TRANSPARENCY DECLARATION

The lead author affirms that this manuscript is an honest, accurate, and transparent account of the study being reported, that no important aspects of the study have been omitted and that any discrepancies from the study as planned (and registered with) have been explained. The reporting of this work is compliant with STROBE guidelines.
